# Early Cognitive Decline? Immediate Recall Deficits Predict Alzheimer's Progression

**DOI:** 10.1002/alz70857_107111

**Published:** 2025-12-26

**Authors:** Sara Fernández Guinea, Palmira González‐Erena, Jennifer Burgos‐Webster, Dennis Trejo

**Affiliations:** ^1^ Universidad Complutense de Madrid, Madrid, Spain; ^2^ Complutense University of Madrid, Madrid, Madrid, Spain; ^3^ Universidad CEU San Pablo, Madrid, Madrid, Spain; ^4^ APANID, Madrid, Madrid, Spain

## Abstract

**Background:**

Identifying cognitive markers for early detection of neurodegenerative disorders is crucial for understanding pathological aging. Given the early impact of information acquisition, consolidation, and retrieval processes in Alzheimer's disease (Jutten et al., 2020), this study aims to determine whether immediate or delayed recall processes at baseline can effectively differentiate between individuals with stable mild cognitive impairment (MCI) and those who convert to Alzheimer's Clinical Syndrome (ACS) after two years.

**Method:**

A cohort of 140 older adults diagnosed with MCI (62.4% female, mean age = 76.27, SD = 5.71) underwent baseline neuropsychological assessment, including the Spanish version of the California Verbal Learning Test (CVLT‐TAVEC), the Rey–Osterrieth Complex Figure Test (ROCF), and Verbal Paired Associates (Wechsler Memory Scale). After a two‐year follow‐up, 24 participants progressed to ACS (MCI‐converters). Binary logistic regression and ROC curve analyses were performed to determine whether immediate or delayed recall measures at baseline were more effective in predicting conversion.

**Result:**

Binary logistic regression revealed that immediate recall performance on both verbal (CVLT‐TAVEC) and visual (ROCF) memory tests significantly predicted conversion to ACS within two years (*p* < 0.001). Participants who later developed ACS showed significantly lower baseline scores on short‐term recall tasks compared to those who remained stable (*p* < 0.001). ROC curve analyses demonstrated that CVLT‐TAVEC immediate recall (AUC = 0.815, *p* < 0.000) was a stronger predictor of conversion than long‐term recall (AUC = 0.804, *p* < 0.000).

**Conclusion:**

These findings highlight immediate consolidation deficits as a potential early marker of prodromal Alzheimer's disease, emphasizing their role in the preclinical trajectory of AD. The results suggest that immediate recall impairments, rather than delayed recall deficits, may better capture the earliest cognitive dysfunctions associated with disease progression. Incorporating short‐term recall measures into clinical assessments could improve the early identification of high‐risk individuals and refine predictive models for AD. Further research is needed to explore the underlying neural mechanisms linking immediate consolidation to Alzheimer's pathology.

